# Temporal machine learning framework for diabetic foot ulcer healing trajectory prediction

**DOI:** 10.1186/s12938-026-01529-2

**Published:** 2026-02-05

**Authors:** Reza Basiri, Asem Saleh, Shehroz S. Khan, Milos R. Popovic

**Affiliations:** 1 KITE Research Institute, Toronto Rehabilitation Institute, University Health Network, 550 University Avenue, Toronto, Ontario M5G 2A2 Canada; 2 Institute of Biomedical Engineering, University of Toronto, 164 College Street, Toronto, Ontario M5S 3G9 Canada; 3 Vascular Surgery, Department of Surgery, Faculty of Medicine, University of Toronto, Toronto, Ontario Canada; 4 College of Engineering and Technology, American University of the Middle East, 54200 Egaila, Kuwait

**Keywords:** Diabetic foot ulcer, Temporal prediction, Machine learning, Healing phase classification, Treatment optimization, Clinical decision support, Longitudinal analysis, ExtraTrees

## Abstract

**Objectives:**

Diabetic foot ulcer management relies predominantly on reactive treatment adjustments based on current wound status. This study developed an accessible machine learning framework using routinely collected clinical metadata (no imaging required) to predict healing phase transitions at the next clinical appointment, enabling proactive treatment planning with an integrated recommendation system.

**Methods:**

Longitudinal data from 268 patients with 329 distinct ulcers across 890 appointments were analyzed. Features (n $=$ 103) including temporal measurements normalized by inter-appointment intervals were engineered. An Extra Trees classifier was optimized via Bayesian hyperparameter tuning with impurity-based feature selection and sequential augmentation to predict three transition categories: favorable, acceptable, or unfavorable. Threefold patient-level cross-validation ensured robust performance estimation.

**Results:**

Feature selection identified 30 essential predictors, achieving 70.9% dimensionality reduction. The optimized classifier demonstrated 78% ± 4% accuracy with balanced category performance (per-class F1 scores: 0.72–0.84) and average AUC of 0.90. Historical phase features dominated predictive importance. The integrated treatment recommendation system achieved 88.7% within-category agreement for offloading prescriptions across all chronicity levels. Dressing recommendations demonstrated chronicity-stratified performance, with match rates declining from 83.7% for acute wounds to 5.6% for very chronic wounds, appropriately reflecting clinical reality that treatment-resistant wounds require individualized therapeutic experimentation.

**Conclusions:**

This framework demonstrates potential for next-appointment trajectory prediction using accessible clinical metadata without specialized imaging, pending prospective validation. The chronicity-dependent recommendation performance appropriately distinguishes wounds amenable to standardized protocols from treatment-resistant cases requiring iterative experimentation.

## Introduction

Diabetic foot ulcers (DFUs) represent one of the most devastating complications of diabetes mellitus, affecting approximately 6.3% of the global diabetic population and serving as the gateway to 85% of all diabetes-related lower extremity amputations [1, 2]. Despite advances in wound care, only 50% of DFUs achieve healing within one year of optimal treatment, with 5-year mortality rates of 30–50% rivalling or exceeding most cancers [3, 4]. The economic burden is equally staggering, with DFU care consuming $9–13 billion annually in the United States alone, representing up to one-third of all diabetes healthcare expenditure [5]. With diabetes prevalence projected to reach 700 million by 2045, the DFU burden will grow exponentially absent transformative interventions [6].

Current clinical practice relies predominantly on reactive classification systems, including Wagner and the University of Texas, to assess present wound severity and guide immediate treatment decisions [7–9]. While these systems effectively stratify current ulcer severity, they fundamentally lack predictive capability for individual patient trajectories. The 2019 International Working Group on the Diabetic Foot (IWGDF) guidelines issued an explicit recommendation against using existing classification systems for individual prognosis, stating that none possess sufficient complexity to predict personalized outcomes [10]. This documented limitation creates a critical clinical gap: clinicians can assess current wound status but are limited in the tools available to forecast the healing trajectory at the next appointment, missing opportunities for proactive interventions.

The ability to predict healing phase at the next clinical appointment, rather than merely assessing current status, would enable clinicians to optimize treatments pre-emptively and potentially prevent deterioration. Recent machine learning (ML) applications in DFU prediction have shown promise, with temporal approaches incorporating sequential visit data achieving promising performances, area under the curve (AUC) of 0.85–0.92 [11, 12]. However, most published studies focus on two-category final healing outcomes rather than a more detailed breakdown of outcomes at next-visit trajectory prediction, fail to address irregular appointment scheduling inherent in real-world wound care, and lack integration with evidence-based treatment recommendation systems based on detailed, realistic classifications [13]. The massive evidence–practice gap in DFU treatment, exemplified by only 2.2% documented offloading use despite strong evidence for its efficacy, further underscores the need for clinical decision support tools that translate guidelines into individualized, data-driven treatment plans [14, 15]. This study introduces an accessible temporal ML framework using routinely collected clinical metadata to forecast next-appointment wound transitions across three clinically-aligned categories (favorable, acceptable, unfavorable) that incorporate wound chronicity thresholds. We developed temporal feature engineering approaches that normalize clinical measurements by inter-appointment intervals, addressing irregular follow-up schedules in real-world practice. This three-category formulation aligns model outputs with clinical decision-making where treatment modifications depend on trajectory direction [11, 16, 17]. The integrated treatment recommendation system combines hierarchical similarity matching with clinical decision rules to suggest offloading and dressing interventions.

This study advances DFU prediction through three principal contributions:

- *Accessible metadata-based prediction*: Achieved 78% transition prediction accuracy using only routinely collected clinical features without wound imaging, demonstrating that practical ML systems can approach the performance of image-intensive approaches while maintaining deployment feasibility in resource-constrained settings.
- *Transition-based formulation*: Formulated healing prediction as transition category classification (favorable, acceptable, unfavorable) rather than direct phase prediction or binary risk stratification (high-risk vs low-risk), aligning outputs with clinical decision-making where treatment modifications depend on trajectory direction.
- *Chronicity-stratified treatment recommendations*: Demonstrated that dressing recommendation performance varies systematically by wound chronicity (acute: 83.7%, very chronic: 5.6%), revealing that low match rates for treatment-resistant cases reflect appropriate clinical reality; these wounds require iterative experimentation rather than algorithmic protocols.

## Related work

The application of ML to DFU prediction has evolved substantially in recent years, with increasing emphasis on temporal modeling approaches that capture healing dynamics across multiple clinical encounters. Early prediction systems focused predominantly on baseline risk stratification and final healing outcomes, while contemporary approaches increasingly address the more clinically actionable challenge of predicting healing trajectories at intermediate timepoints.

Temporal and sequential modeling represents the most directly relevant domain for next-appointment prediction systems. Spinazzola et al. [11] demonstrated that long short-term memory (LSTM) recurrent neural networks analyzing 1766 DFUs across 3–6 visits achieved 80% accuracy and 85% AUC for predicting visit-to-visit healing progression, establishing that temporal models incorporating sequential visit data substantially outperform baseline-only approaches. Their work extracted temporal features including wound depth, area, and tissue status changes across irregular appointment intervals, directly addressing the challenge of variable visit spacing in real-world wound care. However, their approach required specialized wound imaging devices to extract quantitative wound measurements, limiting deployment and integration feasibility in resource-constrained settings or existing clinical setups. Berezo et al. [16] employed gradient-boosted decision trees on longitudinal electronic health records (EHRs) data from over 1.2 million wounds, achieving an AUC of about 0.85 for predicting healing within 4, 8, and 12 weeks from treatment initiation. Their analysis of 187 covariates using SHapley Additive exPlanations (SHAP) feature importance revealed that days in treatment and temporal changes in wound characteristics provided greater predictive value than static patient demographics. Dallmann et al. [12] further validated the superiority of temporal approaches, demonstrating that models incorporating wound dimension changes over the first 4–5 weeks achieved AUC of about 0.90 for predicting 12-week healing trajectories in 620,356 chronic wounds. These studies collectively establish that temporal feature engineering and sequential modeling substantially improve prediction accuracy compared to cross-sectional approaches. However, critical gaps remain: none address next-appointment healing phase transitions that enable preemptive intervention, none incorporate chronicity-aware classification reflecting established wound healing timelines, and none integrate predictions with actionable treatment recommendations.

Comparative evaluations of ML algorithms for DFU prediction have consistently demonstrated strong performance for ensemble methods and decision tree-based approaches. Basiri et al. [18] evaluated multiple ML algorithms including Support Vector Machines, XGBoost, CatBoost, Random Forest (RF), and Neural Networks for classifying DFU healing phases (inflammation (I), proliferation (P), and remodeling (R)). The One-versus-Rest RF approach achieved optimal performance for accessible, laboratory-independent healing phase classification to inform medical triage and treatment selection. Wang et al. [19] evaluated six ML algorithms for predicting hard-to-heal DFUs, with Naïve Bayesian models achieving AUC of 0.86 for identifying percentage area reduction $<50\%$ at 4 weeks, demonstrating practical application for short-term outcome prediction. Shi et al. [20] employed RF methodology on 1,488 patients to develop weighted risk models achieving AUC of 0.93 for DFU onset prediction, with RF feature importance analysis identifying plasma fibrinogen, neutrophil percentage, and hemoglobin as top predictors. The systematic review by Weatherall et al. [21] synthesized evidence from 18 studies on ML methods for DFU classification and prediction, reporting sensitivities ranging from 75 to 98% and accuracies from 64 to 99% across various algorithms including neural networks, decision trees, and ensemble methods. While these comparative studies establish performance benchmarks for DFU prediction, most focus on long-term outcomes or amputation risk rather than the clinically actionable task of predicting next-visit healing phase transitions that enable preemptive treatment adjustment.

Short-term and next-visit outcome prediction represents an emerging paradigm shift toward actionable clinical decision support. Kounas et al. [22] employed hyperspectral imaging across four consecutive visits to predict short-term DFU healing, demonstrating that visit-to-visit changes in periwound oxyhemoglobin measured at early visits achieved 85% sensitivity and specificity for predicting healing. This work exemplifies the clinical value of early-visit measurements for near-term trajectory forecasting rather than waiting for final outcomes. However, the requirement for specialized hyperspectral imaging limits accessibility compared to metadata-based approaches using routinely collected clinical features.

Clinical decision support systems integrating ML prediction models have demonstrated transformative potential for DFU management. Scoping and systematic reviews by Garces et al. [23] and Reifs Jiménez et al. [24] synthesized evidence from over 100 studies demonstrating artificial intelligence applications across screening, risk prediction, severity classification, treatment planning, and diagnostic decision-making in diabetic foot care. While these reviews establish the feasibility of integrating ML-based prediction into clinical workflows, substantial gaps remain in treatment recommendation systems that translate predicted trajectories into evidence-based intervention protocols.

The existing literature demonstrates consistent evidence that temporal features capturing healing dynamics provide superior predictive value compared to baseline characteristics alone, and that ensemble methods and decision tree-based algorithms perform robustly for clinical prediction tasks. However, substantial gaps persist: (1) most studies predict binary final outcomes rather than multi-category next-appointment transitions enabling preemptive intervention; (2) few incorporate chronicity-aware classification aligned with established wound healing timelines, and (3) prediction models rarely integrate with treatment recommendation systems or prioritize accessible metadata over specialized imaging. The present study addresses these gaps through an accessible temporal ML framework for three-category, chronicity-informed transition prediction coupled with an integrated treatment recommendation system.

## Results

### Training optimization

Bayesian optimization of Extra Trees classifier hyperparameters converged on a configuration of 400 estimators with a maximum depth of 60, balancing model complexity with generalization capability. Feature selection used Extra Trees feature importance scores, selecting the top 30 features that achieved optimal predictive performance, representing a 70.9% reduction from the original 103 available features (Table 1). This efficient feature set captured essential aspects of wound healing dynamics while maintaining model interpretability for clinical decision support.

While feature selection reduced model complexity, a complementary approach involving data augmentation was used to increase training samples while preserving temporal causality. The sequential data augmentation expanded the training set from 560 original transition samples to 1582 augmented samples. Ablation analysis confirmed that augmentation improves both classification and calibration performance: without augmentation, average F1 decreased from 0.76 to 0.73, ECE increased from 0.07 to 0.10, and ROC-AUC decreased from 0.90 to 0.87. This demonstrates that the temporal augmentation strategy, which preserves contiguous appointment sequences while expanding the training set, is beneficial for achieving the model’s reported performance.

**Table 1:** Selected features by category and importance ranking. Features ranked by extra trees feature importance

Rank	Feature	Category
1	Phase-adjusted treatment effect	Treatment response
2	Treatment intensity score	Treatment response
3	Appointments to date	Temporal
4	Treatment history length	Historical pattern
5	Cumulative phase duration	Temporal
6	Historical acceptable transitions	Historical pattern
7	Historical proliferative phase count	Historical pattern
8	Appointment interval	Temporal
9	Historical proliferative phase proportion	Historical pattern
10	Days to next appointment	Temporal
11	Phase improvement count	Treatment response
12	Exudate amount consistency	Wound assessment
13	History completeness	Historical pattern
14	Peri-ulcer temperature	Temperature
15	Historical inflammatory phase proportion	Historical pattern
16	Historical inflammatory phase count	Historical pattern
17	Intact skin temperature	Temperature
18	Normalized peri-ulcer temperature	Temperature
19	Wound center temperature	Temperature
20	Exudate appearance	Wound assessment
21	Exudate amount	Wound assessment
22	Healing momentum	Treatment response
23	Wound duration since onset	Temporal
24	Normalized wound center temperature	Temperature
25	Slow healer phenotype	Patient phenotype
26	Fast healer phenotype	Patient phenotype
27	Historical favorable transitions	Historical pattern
28	Appointment interval variability	Temporal
29	Historical mean exudate	Wound assessment
30	Wound severity score	Wound assessment

**Fig. 1:**
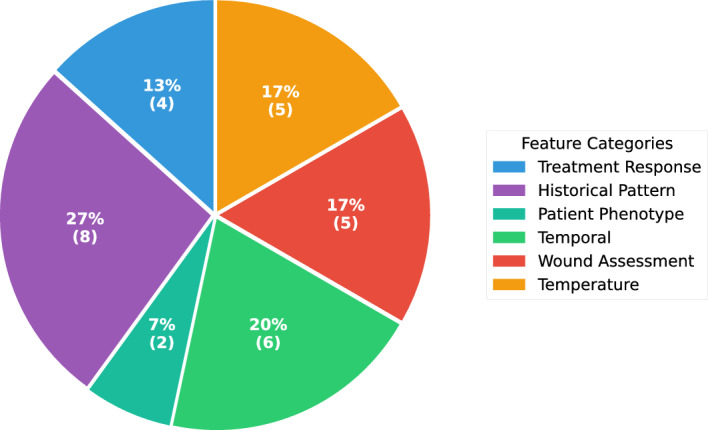
Distribution of selected features by clinical category across six domains: historical pattern, temporal, temperature, wound assessment, treatment response, and patient phenotype

Feature category analysis revealed six clinically meaningful domains (Fig. 1 and Table 1). Historical pattern features (27%) capturing longitudinal healing trajectories comprised the largest category. Temporal features (20%) provided essential appointment timing and scheduling signals. Temperature (17%) and wound assessment (17%) features captured objective wound measurements including thermal patterns and exudate characteristics. Treatment response features (13%) reflected intervention effectiveness, while patient phenotype features (7%) encoded healer clustering assignments.

**Fig. 2:**
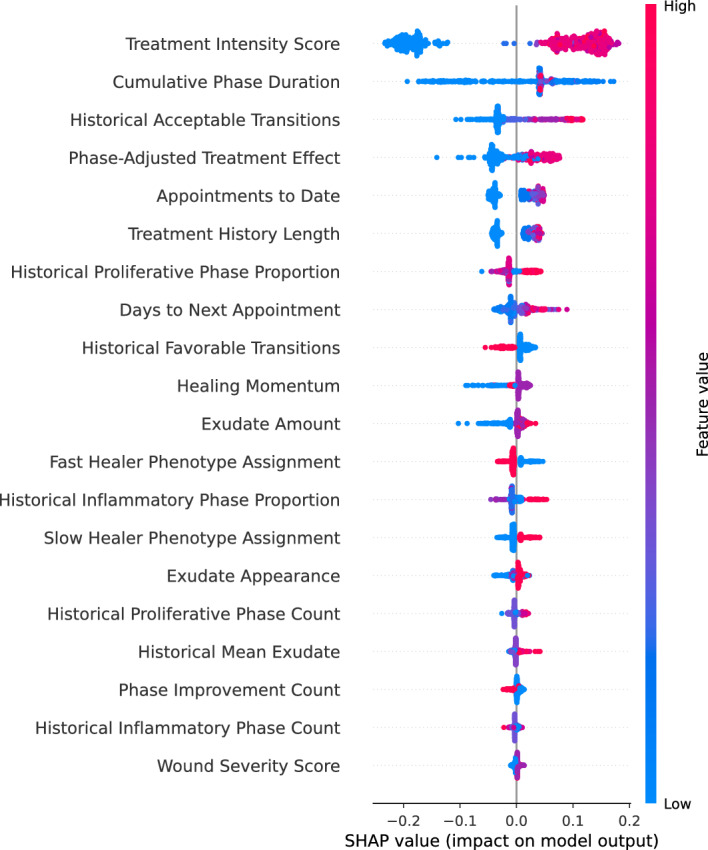
SHAP summary plot for feature importance and directional effects. SHAP summary plot showing feature importance and directional effects on model predictions. Each point represents a single prediction, with color indicating feature value (red = high, blue = low) and horizontal position showing impact on model output. Features are ranked by mean absolute SHAP value. Days to next appointment shows the strongest predictive influence, followed by historical phase proportions and wound characteristics

While the model operated based on the Extra Tree feature importance (Table 1), the SHAP analysis [25] provided additional granular insight into individual feature contributions to model predictions (Fig. 2). Days to next appointment emerged as the dominant predictor, with longer intervals strongly associated with unfavorable transition predictions. Historical I phase proportion showed high impact, where greater time spent in the I phase increased unfavorable outcome likelihood. Treatment-related features including exudate amount and diabetes type demonstrated bidirectional effects depending on their values, enabling clinicians to identify modifiable risk factors for targeted intervention.

### Prediction performance

Threefold patient-level cross-validation of the optimized Extra Trees classifier demonstrated consistent performance across folds for transition category prediction (Table 2). Mean transition accuracy was 78% ± 4% and weighted F1 score 0.76 ± 0.02.

**Table 2:** Threefold cross-validation performance for transition prediction

	Accuracy	F1-weighted	Samples
Mean	0.78	0.76	1582
Std Dev	0.04	0.02	

The coefficient of variation across folds remained low (2.0% to 4.0%), demonstrating stable performance independent of specific patient subset composition.

**Table 3:** Transition category performance metrics

Transition category	Precision	Recall	F1-Score
Unfavorable	0.73	0.89	0.84
Acceptable	0.73	0.71	0.72
Favorable	0.77	0.73	0.75

The ulcer transition category analysis revealed differential performance across transition types (Table 3). Unfavorable transitions (regression or prolonged stagnation) achieved highest F1 score (0.84), demonstrating strong capacity to identify at-risk trajectories requiring clinical intervention. Favorable transitions (healing progression) showed solid performance (F1: 0.75), indicating reliable identification of positive healing trajectories. Acceptable transitions (stable phase maintenance) proved most challenging to classify (F1: 0.72), reflecting the inherent ambiguity of this intermediate category that represents neither clear progression nor deterioration.

**Table 4:** Classification Rates for transition category prediction averaged across three folds

Transition category	TPR% Sensitivity)	FPR%	TNR% (Specificity)	FNR%
Favorable	72.8	8.2	91.8	27.2
Acceptable	70.9	12.2	87.8	29.1
Unfavorable	88.6	12.5	87.5	11.4
**Average**	**77**.**4**	**11**.**0**	**89**.**0**	**22**.**6**

TPR: true positives/total actual positives; FPR: false positives/total actual negatives

TNR: true negatives/total actual negatives; FNR: false negatives/total actual positives

Classification rate analysis (Table 4) revealed balanced performance across transition categories. The 12.5% false positive and 11% false negative rates for one-vs-rest unfavorable classification ensure most high-risk trajectories are correctly identified, and minimizing the risk of them being left untreated.

**Fig. 3:**
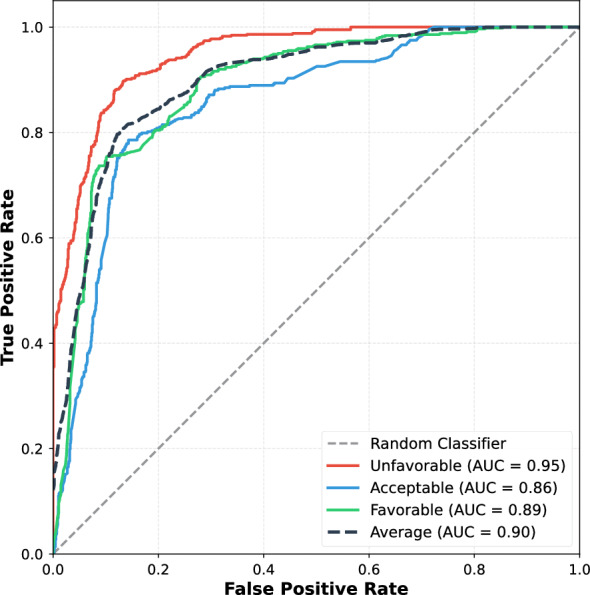
Receiver operating characteristic curves for transition category prediction. Receiver operating characteristic curves demonstrating model discrimination performance for transition category prediction. The model achieved strong discrimination across all categories with average AUC of 0.90. Unfavorable transitions demonstrated highest discrimination (AUC = 0.95), followed by favorable (AUC = 0.89) and acceptable (AUC = 0.87). These discrimination metrics confirm robust classification performance suitable for clinical decision support. AUC: area under the curve

Receiver operating characteristic (ROC) analysis quantified the model’s discrimination capacity for transition category prediction (Fig. 3). The model demonstrated strong overall discrimination with average AUC of 0.90, indicating excellent separation between transition categories across all decision thresholds. Per-class AUC values ranged from 0.87 to 0.95, confirming robust discrimination performance across all categories.

The transition category discrimination revealed clinically meaningful patterns across outcome types. Unfavorable transitions achieved the highest AUC of 0.95, demonstrating excellent capacity to identify deteriorating trajectories requiring clinical intervention. Favorable transitions showed strong discrimination (AUC = 0.89), while acceptable transitions demonstrated robust discrimination (AUC = 0.87) despite representing the most ambiguous intermediate category.

The consistently high discrimination across all categories (all AUC values $>0.87$) validates that the temporal feature engineering successfully captured prognostic patterns enabling accurate next-appointment predictions. These discrimination metrics, combined with the transition classification performance (Tables 2, 3 and 4), establish that the prediction framework provides clinically actionable trajectory forecasts suitable for preemptive treatment optimization.

**Fig. 4:**
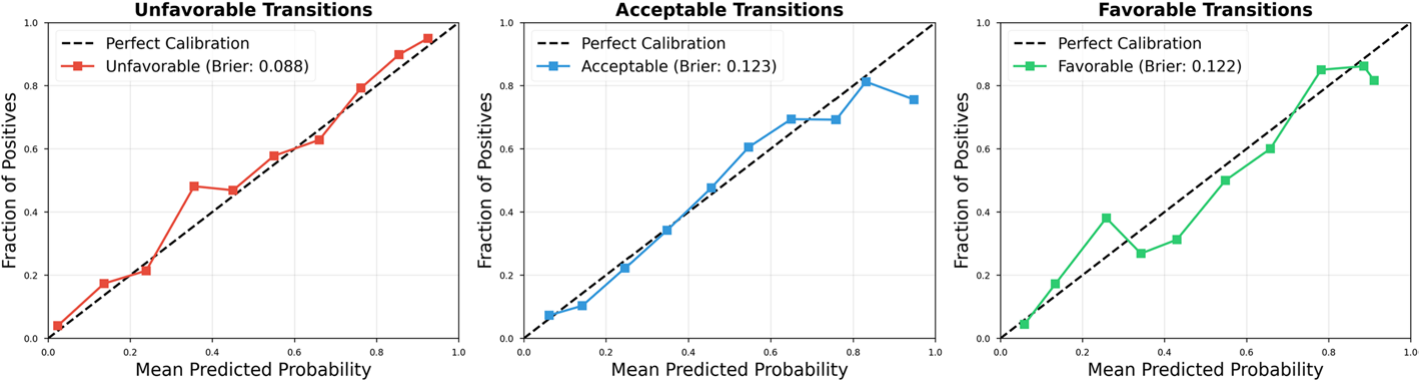
Calibration curves for each transition category showing predicted probability versus observed frequency. Well-calibrated predictions fall along the diagonal (dashed line). Unfavorable and favorable transitions demonstrate strong calibration (ECE = 0.05 and 0.06), while the acceptable category shows moderate calibration error (ECE = 0.09), reflecting the inherent difficulty in predicting this intermediate outcome. ECE: expected calibration error

Calibration analysis assessed the reliability of predicted probabilities for clinical decision-making (Fig. 4). The model demonstrated strong calibration for unfavorable and favorable transitions with expected calibration errors (ECE) of 0.05 and 0.06, respectively, indicating that predicted probabilities closely matched observed frequencies. The acceptable category showed higher calibration error (ECE = 0.09), reflecting the inherent ambiguity of this intermediate class. Overall mean ECE of 0.07 confirms that predicted probabilities provide reliable confidence estimates suitable for clinical risk stratification.

### Treatment recommendation performance

The hierarchical treatment recommendation system underwent evaluation on the validation dataset, assessing both offloading and dressing recommendations against actual clinical prescriptions. The system evaluated 239 test cases from 81 patients, with performance stratified by wound chronicity to identify scenarios where recommendations aligned most closely with specialist clinical judgment.

#### Offloading recommendations

Offloading recommendations demonstrated strong agreement with clinical practice, achieving 88.7% within-category match rate and 62.3% exact match rate (Table 5). The within-category metric considered functionally equivalent offloading modalities as correct (e.g., therapeutic footwear versus modified footwear within the same intensity level), while exact match required identical prescription.

**Table 5:** Offloading recommendation performance

Metric	Value
Within-category match	88.7%
Exact match	62.3%
Mean confidence	0.77

#### Dressing recommendations

Dressing recommendations showed substantial variation in match rates across wound chronicity categories (Table 6), revealing important patterns in when standardized protocols apply versus when individualized approaches become necessary.

**Table 6:** Dressing recommendation performance by wound chronicity

Chronicity category	Match rate
Acute (<90 days)	83.7%
Subacute (90–180 days)	70.1%
Chronic (180–365 days)	67.6%
Very chronic (>365 days)	5.6%

Error analysis of very chronic wounds (>365 days, about n $=$ 40 in the validation set for each fold) revealed systematic divergence between algorithmic recommendations and clinical practice: the model recommended absorptive dressings (Iodosorb: 53.9%, Polysporin: 42.7%) while clinicians predominantly employed standard antiseptic protocols (betadine: 73.0%), suggesting that similarity-based matching identified historically successful advanced treatments while clinicians adopted conservative management for treatment-resistant cases.

### Treatment recommendation examples

This case demonstrates the system’s decision pathway for structural abnormalities requiring enhanced mechanical protection despite acute presentation (Table 7).

**Table 7:** Case 1 patient and wound characteristics

Patient demographics			
Age	58 years	Sex	Female
BMI	20.3 kg/$\hbox {m}^2$	Diabetes type	Type 1
Deformities	Claw toe, hammer toe	Red flags	High deformity

Wound characteristics			
Location	Toes, right foot	Chronicity	Acute (33 days)
Healing phase	Proliferative	Exudate	Minor
Wound severity	Mild	Clinical risk	Moderate
Deformities	Claw toe, hammer toe	Red flags	High deformity

*System recommendations:* Boot or removable cast walker with 0.85 confidence and betadine.

*Actual clinical decision:* Therapeutic footwear (offloading) and betadine (dressing). The clinician selected less intensive offloading than recommended, representing acceptable clinical variation given the acute timeline and mild wound severity. Dressing recommendation showed exact agreement.

*Clinical interpretation:* This case exemplifies the system’s conservative approach to structural deformities, recommending enhanced protection even for mild acute wounds. The offloading discrepancy illustrates appropriate clinical judgment balancing protection against patient mobility and adherence considerations, particularly early in treatment when immediate compliance is critical.

## Discussion

### Next-visit prediction approach

Our transition prediction performance (78% accuracy, 0.90 AUC) demonstrates competitive performance within the emerging next-appointment prediction literature while introducing distinct methodological advantages. Spinazzola et al. [11] achieved 80% accuracy with 85% AUC using LSTM networks on 1766 DFUs monitored across 3–6 visits with only 2 classes (improve vs. worsen), requiring wound imaging to extract features including wound area, depth, and tissue color percentages. In contrast, our system achieves comparable performance using exclusively metadata features routinely documented in EHRs. This distinction enables immediate deployment in diverse clinical settings, including telemedicine, rural clinics, and home healthcare, where specialized wound imaging infrastructure remains unavailable. This metadata-based approach also aligns with emerging standardized wound assessment protocols being implemented globally, such as Bates-Jensen Wound Assessment Tool (BWAT) [26] and Photographic Wound Assessment Tool (PWAT) [27] documentation standards. As these standardized tools capture most features in our selected predictor set, the framework can integrate directly into existing clinical workflows without requiring documentation changes, a critical factor for frontline engagement. The model’s independence from specialized imaging enables immediate deployment while healthcare systems establish a comprehensive wound photography infrastructure.

Our three-category transition formulation (favorable, acceptable, unfavorable) provides greater clinical granularity than binary classification approaches, enabling differentiation between wounds requiring immediate intervention, continued current protocols, or potential treatment de-escalation. While time-to-event models (e.g., Cox regression, survival analysis) offer valuable insights into healing duration distributions, they address a fundamentally different clinical question: estimating when healing occurs rather than what happens at the next visit. Our transition framework provides actionable guidance at each appointment, enabling preemptive treatment adjustment rather than waiting for outcome censoring. The transition category definitions themselves are grounded in established clinical evidence: prolonged inflammation thresholds (>21 days) reflect documented resolution timelines in diabetic wounds [28], while proliferative stagnation thresholds (>42 days) align with the 4–6 week prognostic window validated by Sheehan et al. [29] and Society for Vascular Surgery guidelines [30].

Given this clinically grounded formulation of transition categories, the choice of prediction algorithm becomes critical for translating these temporal thresholds into accurate forecasts. While recurrent neural networks such as LSTM represent a natural architectural choice for sequential medical data, ensemble tree-based methods demonstrate superior performance on structured tabular datasets in small to moderate sample regimes [31, 32]. Given our dataset’s characteristics (1582 augmented transition samples from 560 original transitions) and explicit temporal feature engineering, Extra Trees provided an optimal balance between predictive performance, computational efficiency, and clinical interpretability. Feature importance rankings enable transparent identification of prognostic factors essential for clinical adoption and regulatory approval, whereas deep sequential architectures obscure decision logic within learned weight matrices.

### Treatment recommendation clinical interpretation

The chronicity-dependent dressing recommendation pattern (acute: 83.7%, subacute: 70.1%, chronic: 67.6%, very chronic: 5.6%) should be interpreted as validation of clinical practice rather than as a system failure. The review by Monteiro-Soares et al. [13] found that all 11 evaluated DFU prediction studies exhibited high bias risk, with most failing to acknowledge the fundamental distinction between wounds that respond to standardized protocols and those that require individualized experimentation. Our recommendation system explicitly captures this clinical reality: the 5.6% match rate for very chronic wounds reflects that treatment-resistant cases genuinely need rotating therapeutic trials rather than algorithmic prescriptions. Additionally, the variability in dressing recommendations observed in our results reflects multiple real-world constraints beyond pure clinical judgments. Procurement and formulary limitations substantially influence prescribing patterns, leading to prioritizing product availability over evidence-based selection. Future frameworks should consider mapping evidence-based recommendations to locally available formularies, categorizing dressings by wound presentation (infected, inflamed, wet, dry) and matching these to available product categories (iodine-based, silver-based, non-adherent, exudate management) within institutional constraints.

The greater variability in dressing recommendations compared to offloading protocols reflects fundamental differences in treatment complexity. Offloading decisions follow primarily biomechanical principles with limited modality options (pressure redistribution intensity), whereas dressing selection requires simultaneous optimization across multiple wound bed parameters, including exudate management, infection control, moisture balance, and tissue regeneration [33, 34]. The 12 distinct dressing types in clinical use, versus 4 offloading intensity categories, demonstrate this inherent complexity, with dressing choice necessarily adapting to evolving wound characteristics across healing phases, while offloading principles remain mechanically determined by anatomical and structural factors.

### Feature engineering

The prominence of temporally engineered features in model performance aligns with established evidence that temporal dynamics outperform static baseline assessments in wound healing prediction. Dallmann et al. [12] demonstrated that temporal change features achieved AUC of approximately 0.90 at weeks 4–5, substantially exceeding static measurements. Our implementation of temporal normalization, where features were scaled by inter-appointment intervals, addressed the irregular follow-up schedules characteristic of real-world wound care practice. The emergence of Days to Next Appointment as the top predictor validates that identical wound characteristics yield different trajectory implications depending on reassessment timing: a wound assessed at 7 days versus 42 days represents fundamentally different clinical contexts requiring distinct prognostic interpretation.

Unsupervised clustering identified two distinct patient phenotypes (fast-healer and slow-healer) that captured baseline healing propensity independent of instantaneous wound characteristics. This stratification enabled the model to calibrate predictions by distinguishing typical phenotype-specific variation from genuine clinical deterioration requiring intervention. The approach extends Nie and Zhao’s [35] state transition modeling in intensive care patients, which achieved 93% AUC by encoding previous states as model features. Patient phenotype assignment provided analogous historical context, allowing trajectory deviations to be interpreted relative to expected healing velocity for that subpopulation rather than against population-wide averages that obscure clinically meaningful heterogeneity.

#### Clinical interpretation

Key findings from the integrated prediction and recommendation system:

- *Next-appointment trajectory prediction*: Three-class classification achieved 78% accuracy (weighted F1: 0.76, AUC: 0.90) with strong unfavorable transition detection (F1: 0.84); enabling preemptive treatment modification before deterioration manifests.
- *Treatment recommendations*: Offloading achieved consistent 88.7% match across all chronicity levels; dressing recommendations showed chronicity-dependent performance (acute/subacute: 83.7%/70.1% suitable for algorithmic support; very chronic: 5.6% appropriately flags need for specialist judgment).
- *Clinical implication*: System reliably predicts trajectories and supports routine treatment decisions while identifying high-risk transitions and treatment-resistant wounds requiring individualized clinical expertise

### Limitations and future directions

Several limitations warrant consideration when interpreting these findings. The single-center retrospective design constrains generalizability to other clinical settings with potentially different patient demographics, treatment protocols, or follow-up patterns. The systematic review by Monteiro-Soares et al. [13] identified inadequate sample sizes (only 2/11 studies achieved >200 events-per-variable threshold) and lack of external validation as pervasive methodological deficiencies in DFU prediction research. Our 268-patient cohort, while reasonable for initial model development, requires multi-site validation to establish robustness across healthcare systems. External validation on public datasets remains challenging as existing repositories such as DFUC2024 [36] contain wound images without the clinical metadata essential to our approach; to our knowledge, no publicly available DFU dataset includes the longitudinal clinical features required for metadata-based trajectory prediction.

The severe class imbalance, particularly for remodeling phase (13.3% prevalence) and phase regression transitions (12.9%), limits model reliability for rare but clinically critical events despite resampling. Multiple studies [37, 38] warn that resampling methods can improve discrimination metrics while harming probability calibration, a concern amplified by our focus on accuracy and F1-score. Preliminary calibration analysis revealed that the acceptable transition category exhibited higher calibration error than favorable or unfavorable categories, reflecting the inherent ambiguity of this intermediate class that represents neither clear progression nor deterioration. Future work should incorporate additional datasets and methods [39, 40] to minimize the class imbalance and improve probability calibration.

Our feature set, while comprehensive for routinely collected metadata, excludes several domains with potential prognostic value: wound imaging characteristics, microbiology results, vascular assessment parameters, and social determinants affecting treatment adherence and follow-up consistency. The dataset lacks both quantitative vascular measures (ankle brachial index, toe pressures, arterial Doppler studies) and clinical vascular examination findings (pulse assessment, capillary refill). This exclusion was intentional. The research dataset was designed to evaluate predictive performance using only universally accessible clinical metadata, reflecting real-world constraints in primary care clinics, community health centers, and telehealth consultations where formal vascular diagnostic testing may be unavailable due to equipment costs, staffing constraints, or care delivery modality. The robust model performance demonstrates that clinically meaningful risk stratification remains feasible without these specialized assessments, establishing a baseline against which future studies can quantify the incremental predictive value of incorporating such parameters. Nevertheless, vascular perfusion fundamentally determines both healing potential and treatment urgency in clinical practice. Future implementations in facilities with diagnostic capabilities should incorporate formal vascular assessment data to investigate whether these measurements enhance prediction accuracy beyond the metadata-driven approach validated here. Additionally, the model assumes prescribed treatments continue unchanged until the next appointment, which may not reflect variable patient adherence patterns observed in real-world clinical settings.

The modest dressing recommendation match rates reflect fundamental challenges in wound care decision-making, including individualized considerations (exudate management, infection control, patient sensitivities, cost constraints, formulary limitations) and limited comparative effectiveness evidence across dressing types and wound conditions. The observed divergence for very chronic wounds, demonstrates that treatment-resistant cases require clinical judgment informed by longitudinal response patterns and practical constraints (cost, patient tolerance, formulary availability) not captured in single-appointment feature sets. Clinical documentation rarely captures systematic treatment response or selection rationale. Future research should prioritize the prospective collection of dressing-specific outcome data, with documented clinical reasoning, to improve recommendation algorithms. Critically, the predictions and recommendations generated by this framework represent ML outputs that have not been clinically validated through prospective studies; implementation in clinical practice would require careful evaluation of prediction accuracy in real-world settings and assessment of impact on patient outcomes before routine deployment. The extensive clinical applications, coupled with large-scale language models [41, 42], can provide a promising and innovative enhancement to DFU care and treatment. Regarding regulatory considerations, clinical deployment would require appropriate Software as a Medical Device classification and compliance with applicable regulations; the framework is designed as clinical decision support requiring physician oversight rather than autonomous diagnostic capability.

## Conclusion

This study presents a temporal ML framework with potential to transform DFU management from reactive assessment to proactive prediction. By forecasting healing transitions at the next appointment with 78% accuracy and 0.90 AUC, our approach may enable clinicians to optimize treatments before adverse trajectories manifest, pending prospective clinical validation. The integrated treatment recommendation system achieved 88.7% offloading agreement, with chronicity-dependent dressing performance appropriately reflecting that treatment-resistant wounds require individualized experimentation rather than algorithmic protocols.

This work demonstrates that practical next-appointment prediction systems can approach the performance of imaging-intensive approaches while maintaining deployment feasibility through reliance on accessible clinical metadata. The transition-based formulation provides clinicians with actionable trajectory information at each encounter, potentially enabling preemptive treatment modification before adverse transitions occur. However, these findings require validation through prospective clinical studies before implementation in routine practice.

## Methods

### Study design and ethical considerations

This retrospective cohort study analyzed longitudinal DFU healing trajectories to develop predictive models for next-appointment healing phase transition classification. The dataset was collected with approvals from the Conjoint Health Research Ethics Board of the University of Calgary (#21-1052) and Research Ethics Board of the University Health Network (#21-5352). All patient data were anonymized prior to analysis.

### Dataset description

This study utilized the Zivot dataset [43], a comprehensive DFU clinical dataset collected at a specialized wound care center in Alberta, Canada. This clinic implements the Toe and Flow model which has been shown to reduce DFU complications significantly [44, 45]. The dataset comprised 890 appointment records from 268 unique patients with 329 distinct DFUs.

**Table 8:** Dataset characteristics and structure

Characteristic	Value	Description
*Dataset overview*		
Total records	890	Appointment records
Unique patients	268	Individual patients
Unique wounds	329	Distinct DFUs
Total raw features	72	56 numerical, 16 categorical
Appointments per patient	$3.3 \pm 3.2$	Mean ± SD (range: 1–17)
Appointments per wound	$2.7 \pm 2.2$	Mean ± SD
Missing data	4.57%	Overall missingness
*Patient demographics*		
Age	$62.3 \pm 11.7$ years	Mean ± SD (range: 27–93)
Male gender	212 (79.1%)	Male predominance
Patients $>60$ years	147 (54.9%)	Elderly population
Patients $>70$ years	64 (23.9%)	Advanced age
*Wound chronicity distribution*		
Acute ($<90$ days)	96 (30.8%)	Recent onset
Subacute (90–180 days)	51 (16.3%)	Intermediate duration
Chronic (180–365 days)	46 (14.7%)	Prolonged healing
Very chronic ($>365$ days)	119 (38.1%)	Treatment resistant
Mean duration	$565.3 \pm 932.5$ days	At presentation
Excluded	17	Missing onset or dressing type
*Healing phase distribution*		
Inflammatory (I)	276 (31.0%)	Initial phase
Proliferative (P)	495 (55.7%)	Active healing
Remodeling (R)	118 (13.3%)	Final maturation
*Phase transitions (n = 560)*		
Improvement	123 (22.0%)	Phase progression
Stable	365 (65.2%)	No phase change
Regression	72 (12.9%)	Phase deterioration
*Treatment protocols*		
Unique offloading types	5	Treatment variety
Unique dressing types	12	Treatment variety
*Temporal patterns*		
Appointment interval	$33.4 \pm 36.0$ days	Mean ± SD
Median interval	21 days	Typical follow-up
Treatment duration	$94.6 \pm 78.2$ days	Total course
Appointments $\le 14$ days	203 (26.6%)	Frequent monitoring
Appointments $>28$ days	188 (24.6%)	Extended intervals
*Data structure*		
Single appointment wounds	131	Excluded from analysis
Multiple appointment wounds	198	Included for longitudinal analysis
Wounds with $\ge 3$ visits	127 (38.6%)	Extended follow-up

Table 8 summarizes the dataset characteristics. The study population demographics align with established DFU epidemiology [1], showing male predominance and concentration in elderly patients.

### Model selection

The selection of the Extra Trees classifier [46] for next-appointment healing phase prediction was informed by our previous comprehensive evaluation demonstrating RF’s superior performance for DFU metadata classification [18]. Extra Trees represents a natural evolution of the RF algorithm, introducing additional randomization during tree construction to improve generalization and reduce overfitting risk in longitudinal clinical data. The key algorithmic distinction lies in split point selection: while RF evaluates multiple candidate thresholds for each feature to identify optimal splits, Extra Trees randomly selects split thresholds, then chooses the best among these random candidates. This additional stochasticity serves multiple purposes for our application: (1) reduced computational complexity enabling faster training on augmented datasets; (2) decreased correlation between individual trees improving ensemble diversity; and (3) enhanced robustness to noisy or missing clinical measurements common in real-world wound care settings [46]. For Extra Trees classifier hyperparameter optimization, 2000 iterations of Bayesian optimization with Gaussian Process surrogate models were employed to efficiently explore the high-dimensional parameter space while minimizing computational cost. The optimized configuration of 400 estimator trees with maximum depth 60 reflects ensemble learning principles: numerous weak learners reduce prediction variance through averaging, while the nominal maximum depth serves as an upper bound rarely reached in practice due to minimum samples per leaf constraints that adaptively limit tree complexity based on available data [31].

The classifier processed data in terms of features and target classes. The target classes were formulated into transition category classification, grouping phase changes into clinically meaningful outcomes based on established wound healing physiology. **Favorable** outcomes encompassed healing progression (I$\rightarrow$P, I$\rightarrow$R, P$\rightarrow$R) occurring within expected healing timeframes, or maintained remodeling (R$\rightarrow$R), representing clear healing advancement [28, 47]. **Acceptable** outcomes included stable proliferation (P$\rightarrow$P $\le$42 days), recognizing that wound healing literature supports a 4-week threshold for identifying stagnation, with wounds failing to achieve 50% area reduction by 4 weeks demonstrating 91% negative predictive value for complete healing [29]. Additionally, inflammatory persistence (I$\rightarrow$I $\le$21 days) was considered acceptable, as normal inflammatory resolution occurs within 7–14 days in acute wounds but is commonly extended in DFUs [28]. **Unfavorable** outcomes comprised phase regressions (P$\rightarrow$I, R$\rightarrow$I, R$\rightarrow$P) at any interval, prolonged inflammation (I$\rightarrow$I >21 days), where wounds remained in the inflammatory phase beyond the expected 2–3 week resolution period [28], or prolonged proliferation (P$\rightarrow$P >42 days), where wounds exhibited stagnation beyond the evidence-based 6-week threshold recommended by Society for Vascular Surgery guidelines [30]. This transition-based formulation aligns prediction with clinical decision-making, where treatment modifications depend on both trajectory direction and temporal progression rather than instantaneous phase assessment [2]. For feature selection, an impurity-based importance threshold approach using the Extra Trees classifier’s native feature importance was incorporated directly into the Bayesian optimization process to select the essential features for achieving the highest performance.

### Data preprocessing and feature engineering

Overall, the candidate feature pool encompassed multiple domains reflecting different aspects of wound healing:

The candidate feature pool encompassed temporal features (appointment intervals, days since onset, healing momentum), wound characteristics (exudate properties, dimensions, temperature, tunneling), treatment features (dressing types, offloading modalities, consistency metrics), patient factors (age, body mass index, comorbidities, mobility risk), anatomical features (location, deformities, foot scores), peri-ulcer conditions (erythema, edema, pallor, maceration), and engineered features (historical aggregations, cluster assignments, treatment interactions). This comprehensive feature set captured both static patient characteristics and dynamic healing indicators, enabling the model to learn complex patterns across multiple timescales and physiological domains.

#### Temporal feature engineering

To capture healing dynamics normalized by irregular appointment intervals, we engineered comprehensive temporal features addressing the real-world challenge of variable follow-up schedules. Historical aggregation features computed mean, standard deviation, minimum, and maximum values across all previous appointments for continuous variables, providing longitudinal context. Phase transition metrics quantified healing trajectory patterns through counts of improvements (transitions to higher phases), regressions (transitions to lower phases), and stability periods. Our engineered healing momentum feature captured the recent trajectory dynamics:

$$\begin{aligned} \text {Healing Momentum} = \frac{1}{n}\sum _{i=1}^{n} w_i \cdot \Delta \text {Phase}_i, \end{aligned}$$

where $w_i$ represents recency weights emphasizing recent transitions over historical patterns, and *n* denotes the number of recent appointments considered.

#### Patient clustering for phenotype identification

Unsupervised k-means clustering (k = 2) on the training dataset identified distinct patient healing phenotypes based on four key metrics: average healing phase across appointments, healing velocity (phase change per day), phase stability (standard deviation of phases), and treatment responsiveness (correlation between treatment changes and phase improvements). Features were standardized prior to clustering to ensure equal contribution across different scales. This clustering revealed two phenotypes with distinct healing capacities (Fast: 42 ± 18 days vs Slow: 156 ± 72 days, p<0.001) with healing velocity of 0.012 ± 0.008 vs. 0.003 ± 0.005 phase units/day. Cluster assignments were incorporated as binary features (Fast Healer, Slow Healer), enabling phenotype-specific prediction patterns. To avoid data leakage, the clustering was performed on the training data only, with the resulting cluster models subsequently applied to the validation data to prevent information leakage.

#### Treatment feature engineering

Treatment-related features captured both current interventions and historical treatment patterns. Composite features aggregated multiple treatment modalities into single metrics: deformity severity combined structural abnormalities using clinical weights (Charcot arthropathy:5 points, claw or hammer toes:2 points each, bunion deformities:1 point), reflecting relative clinical impact on offloading requirements. Mobility risk combined multiple indicators, including advanced age (>70 years), elevated weight (>90 kg), sensory neuropathy presence, and extreme age (>80 years). Moisture management needs quantified wound exudate challenges through a weighted combination of exudate amount, maceration presence (doubled weight), edema, and wound tunneling (doubled weight). Infection risk similarly aggregated warning signs including odor presence (doubled weight), erythema, and pallor indicators.

The treatment intensity score summed all active offloading modalities and dressing complexity, providing an overall measure of intervention aggressiveness. Historical treatment consistency scores calculated the proportion of previous appointments maintaining the same dressing or offloading approach, capturing adherence and protocol stability. These composite features captured complex clinical presentations that individual measurements might miss, enabling the model to recognize syndrome patterns rather than isolated findings.

#### Data augmentation strategy

To address limited longitudinal data while maintaining temporal integrity, a safe sequential appointment combination augmentation strategy was implemented on the training set only. Importantly, validation samples remained unaugmented to ensure performance metrics reflect true generalization capability. This approach generates multiple training samples for each target appointment by systematically varying the length of historical context while preserving temporal continuity. For a patient with appointments A1 through A5, prediction of appointment A4 yields three training samples:

- Complete history: [A1, A2, A3] $$\rightarrow $$ A4
- Recent history: [A2, A3] $$\rightarrow $$ A4
- Immediate history: [A3] $$\rightarrow $$ A4

This methodology ensures contiguous sequences are maintained, preserving temporal flow while expanding the training set. It deliberately avoids non-contiguous sequences (e.g., [A1, A3] $\rightarrow$ A5) that could introduce spurious patterns and violate temporal causality assumptions critical for clinical validity.

Through this sequential data augmentation, the training set was expanded from 560 original transition samples to 1582 augmented samples, with each sample constructed by pairing features from appointment *t* with the healing phase outcome at appointment $t+1$. This temporal augmentation strategy differs fundamentally from feature selection (dimensionality reduction), through which the feature space was independently reduced from 103 to 30 predictors. While data augmentation increases training samples and preserves temporal causality, feature selection reduces model complexity while maintaining predictive performance, complementary approaches applied during different stages of model development.

### Cross-validation strategy

Model training employed threefold patient-level stratified cross-validation to ensure robust performance estimation while preventing information leakage. Critically, all appointments from a single patient remained within the same fold, preventing the model from learning patient-specific patterns that would not generalize to new patients encountered in clinical deployment. Missing values were addressed using k-Nearest Neighbors imputation with k $=$ 5 neighbors, leveraging similarity between patients to estimate missing measurements.

### Performance metrics

Model performance was evaluated using multiple complementary metrics that address different aspects of transition-prediction quality.

- *Balanced accuracy (or accuracy in this study):* The average of per-class recall values, providing equal weight to each transition category (favorable, acceptable, unfavorable) regardless of prevalence. This metric addresses class imbalance by ensuring that performance on rare transitions receives equal consideration to common transitions [48].
- *Precision:* The proportion of true positives among all positive predictions for each class, indicating how many of the predicted transitions were correct. Calculated per class and averaged across categories.
- *Recall (Sensitivity):* The proportion of actual positive cases correctly identified by the model for each class, indicating the model’s ability to detect all instances of each transition category.
- *Weighted F1 Score:* The weighted average of per-class F1 scores, balancing precision and recall while accounting for class frequencies in the validation set.
- *ROC-AUC:* The probability that the classifier ranks a randomly chosen positive instance higher than a randomly chosen negative instance, computed using the one-versus-rest strategy for multiclass classification. Reported AUC values represent the mean of per-class AUCs. This metric evaluates the model’s discriminative ability across all probability thresholds [49].
- *Expected calibration error (ECE):* Measures the discrepancy between predicted probabilities and observed frequencies across probability bins. Lower ECE values indicate better calibrated probability estimates, essential for clinical risk stratification where probability magnitudes guide decision-making.
- *Confusion matrix:* Complete cross-tabulation of true versus predicted transition categories, revealing systematic patterns in misclassification.
- *SHAP (SHapley Additive exPlanations):* Model-agnostic interpretability method that quantifies each feature’s contribution to individual predictions [25]. SHAP values enable identification of factors driving the predictions.

### Treatment recommender approach

Following next-appointment healing phase prediction, a hierarchical treatment recommendation system was developed to suggest optimal offloading and dressing interventions. The system combines evidence-based clinical decision rules with similarity-based matching from a curated database of successful treatment outcomes, providing personalized recommendations informed by both established protocols and historical case patterns.

The recommendation system employed case-based reasoning to match patient presentations against a database of historically successful cases, defined as those demonstrating favorable phase transitions (I$\rightarrow$P, P$\rightarrow$R) or maintenance of advanced healing phases across multiple appointments.

Features were organized into three clinical decision tiers with differential weighting: critical tier encompassing treatment-determinative factors including healing phase, structural deformities, and anatomical risk; important tier comprising wound severity, patient demographics, and chronicity; and refining tier containing individualization modifiers. For offloading, critical factors included Charcot arthropathy and deformity severity; for dressing selection, wound phase, chronicity, and prior treatment response.

Similarity quantification employed a hierarchical adaptation of established case-based reasoning weighted similarity measures [50, 51], modified to accommodate multi-tier clinical decision structures unique to the DFU cases:

$$\begin{aligned} \text {Similarity}(Q, C) = \frac{\sum _{t} w_t \times \sum _{f \in F_t} \text {sim}_f(q_f, c_f)}{\sum _{t} w_t \times |F_t|}, \end{aligned}$$

where $w_t$ represents tier weight, $F_t$ denotes features within tier *t*, and $\text {sim}_f$ quantifies local similarity (binary for categorical features, normalized difference for continuous features). This approach ensured fundamental characteristics determined primary treatment selection while secondary factors provided refinement.

The treatment recommendation system underwent evaluation through retrospective validation, comparing recommended treatments against actual prescribed treatments in the validation dataset. For offloading, two match metrics were computed: exact match required identical device prescription (e.g., therapeutic footwear, crutches), while within-category match allowed recommendations within one intensity level on a four-point ordinal scale (0=no offloading, 1=low-intensity such as therapeutic footwear, 2=moderate-intensity such as removable walker, 3=high-intensity such as total contact cast or assistive devices). Offloading intensity categorization was validated by two podiatric surgeons at the data collection site to ensure clinical equivalence between adjacent levels. For dressings, only exact match was computed given categorical rather than ordinal groupings.

### Predictor and recommender integration

The treatment recommender operated sequentially with the healing phase predictor to enable proactive treatment optimization. After predicting next-appointment transition category, unfavorable predictions triggered treatment recommendation generation. This integrated approach enabled preemptive treatment modification based on predicted trajectories rather than reactive adjustment after deterioration, potentially preventing adverse transitions through timely intervention optimization. The system’s dual output of predictions and recommendations provided comprehensive decision support, addressing both the question of what will happen and what should be done about it.

## Data Availability

The dataset analyzed during the current study cannot be made publicly available due to patient privacy regulations, institutional data sharing agreements, and ethical approval restrictions from the Conjoint Health Research Ethics Board of the University of Calgary (#21-1052) and Research Ethics Board of the University Health Network (#21-5352). The dataset contains sensitive clinical information from diabetic foot ulcer patients that, even when anonymized, could potentially be re-identified given the detailed longitudinal nature of the wound healing trajectories and the relatively small specialized patient population. Access to the data may be granted for legitimate research purposes through a formal data-sharing agreement. Researchers interested in collaborative access should contact the corresponding author (R.B.) at reza.basiri@mail.utoronto.ca with a detailed research proposal. Any data sharing will require approval from the relevant institutional review boards and execution of appropriate data use agreements, ensuring compliance with privacy regulations. The analysis code and trained models supporting this study’s findings will be made available at a GitHub repository upon manuscript acceptance. Summary statistics and aggregated results supporting the conclusions of this article are included within the manuscript and its tables.

## Code availability

The analysis code and trained models are available at https://github.com/rezabasiri/DFUHealing_Trajectory_Prediction.
